# Draft Genome Sequence of Thermophilic *Exiguobacterium* sp. Strain JLM-2, Isolated from Deep-Sea Ferromanganese Nodules

**DOI:** 10.1128/genomeA.00794-15

**Published:** 2015-07-23

**Authors:** De-Chao Zhang, Yan-Xia Liu, Ying-Yi Huo, Xue-Wei Xu, Xin-Zheng Li

**Affiliations:** a Institute of Oceanology, Chinese Academy of Sciences, Qingdao, China; b Laboratory of Marine Ecosystem and Biogeochemistry, Second Institute of Oceanography, State Oceanic Administration, Hangzhou, China

## Abstract

*Exiguobacterium* sp. strain JLM-2 is a thermophilic bacterium isolated from deep-sea ferromanganese (FeMn) nodules. The estimated genome of this strain is 2.9 Mb, with a G+C content of 48.32%. It has a novel circular 15,570-bp plasmid. The draft genome sequence may provide useful information about Mn-microbe interactions and the genetic basis for tolerance to environment stresses.

## GENOME ANNOUNCEMENT

The genus *Exiguobacterium* was first described in 1983 by Collins et al. (1), and the species formerly described as *Brevibacterium acetylicum incertae sedis* was included in the genus *Exiguobacterium* as *E. acetylicum* in 1994 (2). *Exiguobacterium* spp. have been isolated from, or molecularly detected in, many extreme environments, such as Antarctic ice (3), Himalayan glaciers (4), Siberian permafrost (5), deep-sea hydrothermal vents (6), and hot/hyperalkaline springs (7, 8).

The isolate *Exiguobacterium* sp. strain JLM-2 was isolated from deep-sea FeMn nodule samples, which were collected at a water depth of 3,573 m using Jiaolong’s operated submersible loaded on the Xiangyanghong 09 scientific exploration ship from Jiaolong Seamount’s dormant hydrothermal vent, South China Sea, during the first experimental application cruise on 3 July 2013. The concentrations of MnO and Fe_2_O_3_ in the FeMn nodules were 40% and 10%, respectively, by inductively coupled plasma-mass spectrometry (ICP-MS).

*Exiguobacterium* sp. JLM-2 is a Gram-positive extracellular amylase-producing bacterium that can grow at 4 to 53°C and has resistance to Mn^2+^ (220 mM). Based on 16S rRNA gene phylogeny, the isolate is closely related to group II within the *Bacillales* family XII *incertae sedis*, *Firmicutes* (7).

Genomic DNA was extracted and sequenced using the Illumina HiSeq 2500 platform and assembled using Velvet version 1.0 (9). This assembly generated 23 contigs, with an *N*_50_ length of 343,188 bp and an average length of 126,112 bp, which were assembled into 12 scaffolds, with an *N*_50_ length of 2,092,945 bp and an average length of 241,796 bp. The draft genome of *Exiguobacterium* sp. JLM-2 consists of a total 2,998 open reading frames (ORFs). Its genome has a novel circular 15,570-bp plasmid with 19 ORFs. The draft genome is 2.9 Mb, with a G+C content of 48.32%.

### Nucleotide sequence accession number.

This whole-genome shotgun project has been deposited at DDBJ/EMBL/GenBank under the accession no. LDNY00000000.
